# ICAM-2 facilitates luminal interactions between neutrophils and endothelial cells *in vivo*

**DOI:** 10.1242/jcs.137463

**Published:** 2014-02-01

**Authors:** Krishma Halai, James Whiteford, Bin Ma, Sussan Nourshargh, Abigail Woodfin

**Affiliations:** William Harvey Research Institute, Barts and the London School of Medicine and Dentistry, Queen Mary University of London, Charterhouse Square, London EC1M 6BQ, UK

**Keywords:** Intercellular adhesion molecule 2, ICAM-2, Leukocyte, Neutrophil, Extravasation, Intravascular crawling

## Abstract

Intercellular adhesion molecule 2 (ICAM-2) is expressed on endothelial cells (ECs) and supports neutrophil extravasation. However, the full details of its role remain unknown, and the present study investigates the functional mechanisms of ICAM-2 in neutrophil–endothelial-cell interactions. Our initial studies showed expression of ICAM-2 at both EC junctions and on the EC body. In line with the observed expression profile analysis of neutrophil–vessel-wall interactions using real-time *in vivo* confocal microscopy identified numerous functional roles for ICAM-2 within the vascular lumen and at the stage of neutrophil extravasation. Functional or genetic blockade of ICAM-2 significantly reduced neutrophil crawling velocity, increased frequency of crawling with a disrupted stop-start profile, and prolonged interaction of neutrophils with EC junctions prior to transendothelial cell migration (TEM), collectively resulting in significantly reduced extravasation. Pharmacological blockade of the leukocyte integrin MAC-1 indicated that some ICAM-2-dependent functions might be mediated through ligation of this integrin. These findings highlight novel roles for ICAM-2 in mediating luminal neutrophil crawling and the effect on subsequent levels of extravasation.

## INTRODUCTION

Leukocyte extravasation from blood vessels into surrounding tissues is a crucial component of physiological and pathological inflammation. In recent years, many studies, particularly those employing imaging modalities, have significantly furthered our understanding of the profile and mechanisms that support this process. Extravasation occurs through a series of distinct stages, with each step being tightly regulated through defined molecular interactions between leukocytes and components of the venular wall. Specifically, in order to enter the extravascular tissue, migrating leukocytes must first breach endothelial cells (ECs) that line the vessel lumen and then cross the perivascular pericyte layer embedded within the dense matrix protein structure of the vascular basement membrane (Nourshargh et al., 2010). Extravasation begins with tethering and rolling of leukocytes along the luminal endothelium followed by slow rolling that enables the establishment of a stronger interaction between leukocytes and ECs, responses largely triggered and mediated by surface-expressed chemokines (Ley et al., 2007). To initiate extravasation, firmly adherent cells polarise and crawl along the luminal aspect of endothelial cells, seeking to identify permissive sites in the venular wall.

The molecular interactions that mediate the above adhesion cascade have been studied extensively and many aspects are now well characterised. As well as better understanding of the initial luminal responses of rolling and crawling, there is now an abundance of information regarding the role of numerous EC junctional proteins, such as platelet endothelial cell adhesion molecule (PECAM-1), junctional adhesion molecule (JAM)-A, JAM-C, CD99, endothelial cell-selective adhesion molecule (ESAM) and intercellular adhesion molecule 2 (ICAM-2, also known as CD102), in regulation of leukocyte transendothelial migration (TEM) (Ley et al., 2007; Nourshargh et al., 2010). Despite this significant progress, understanding the details of the dynamics of luminal leukocyte–vessel-wall interactions *in vivo* requires further investigation, and there is a need for more in-depth analysis of the functions role of key adhesion molecules. One EC adhesion molecule that has not been extensively studied is ICAM-2.

ICAM-2 is a 55–65 kDa β2 integrin ligand member of the immunoglobulin superfamily that is constitutively expressed on ECs and has also been reported to be expressed on a number of leukocyte subsets, including monocytes, eosinophils, T and B lymphocytes and neutrophils (de Fougerolles et al., 1991; Sundd et al., 2012). On ECs the expression of ICAM-2 is not generally considered to be regulated during inflammation (de Fougerolles et al., 1991), although a limited number of *in vitro* studies have indicated that its expression is reduced post cytokine stimulation in cultured ECs (McLaughlin et al., 1998). ICAM-2 ligands have been identified as the β2 integrin-containing lymphocyte function-associated antigen 1 (LFA-1) (Li et al., 1993; Staunton et al., 1989) and macrophage-1 antigen (MAC-1) (Xie et al., 1995), and DC-SIGN (Geijtenbeek et al., 2000). ICAM-2 forms intracellular interactions with ezrin (Heiska et al., 1996; Yonemura et al., 1998) and α-actinin (Heiska et al., 1996), and has also been demonstrated to bind in a homophilic manner in trans, a response implicated in EC tube formation and angiogenesis (Huang et al., 2005).

In contrast to many EC junctional proteins, ICAM-2 has not been extensively investigated in relation to leukocyte extravasation; however, a number of studies have demonstrated a role for this molecule in recruitment of lymphocytes (Boscacci et al., 2010; Lehmann et al., 2003; Steiner et al., 2010), neutrophils (Huang et al., 2006; Issekutz et al., 1999), monocytes (Schenkel et al., 2004), eosinophils (Gerwin et al., 1999) and dendritic cells (Geijtenbeek et al., 2000), although few have sought to identify the mechanisms underlying the observed roles for ICAM-2. Of those that have addressed this issue, findings indicate that EC ICAM-2 is involved in monocyte ‘locomotion’ on human umbilical vein endothelial cells (HUVECs) through its interaction with β2 integrins (Schenkel et al., 2004), and supports lymphocyte polarisation and crawling on a blood–brain barrier model *in vitro* (Steiner et al., 2010). Few studies, however, have addressed the functional profile of ICAM-2 and the mechanisms through which ICAM-2 supports leukocyte extravasation *in vivo*. Previous work from our laboratory has provided direct *in vivo* evidence for the involvement of EC ICAM-2 in the early phases of neutrophil extravasation (Huang et al., 2006; Woodfin et al., 2009), although details of this process remain unknown. To extend these findings in the present study we have used confocal intravital microscopy (IVM) to conduct an in-depth analysis of the functional role of ICAM-2 in the dynamics of neutrophil–vessel-wall interactions *in vivo*.

We used ICAM-2-deficient mice and pharmacological blockade of ICAM-2, and found a key role for ICAM-2 in the regulation of neutrophil crawling dynamics, and we describe mechanisms that are crucial in supporting neutrophil migration to EC junctions and a possible role during the initiation of an efficient neutrophil TEM response. The results of this study suggest that these ICAM-2-dependent responses may be at least partly mediated through ICAM-2–MAC-1 interactions. Collectively the present work provides evidence for previously unreported functions of ICAM-2 in supporting luminal neutrophil–vessel-wall interactions *in vivo*, responses that can account for the role of ICAM-2 in mediating neutrophil extravasation in acute inflammatory responses.

## RESULTS

### ICAM-2 is expressed at endothelial cell junctions and on the endothelial cell body

**Fig. 1.**
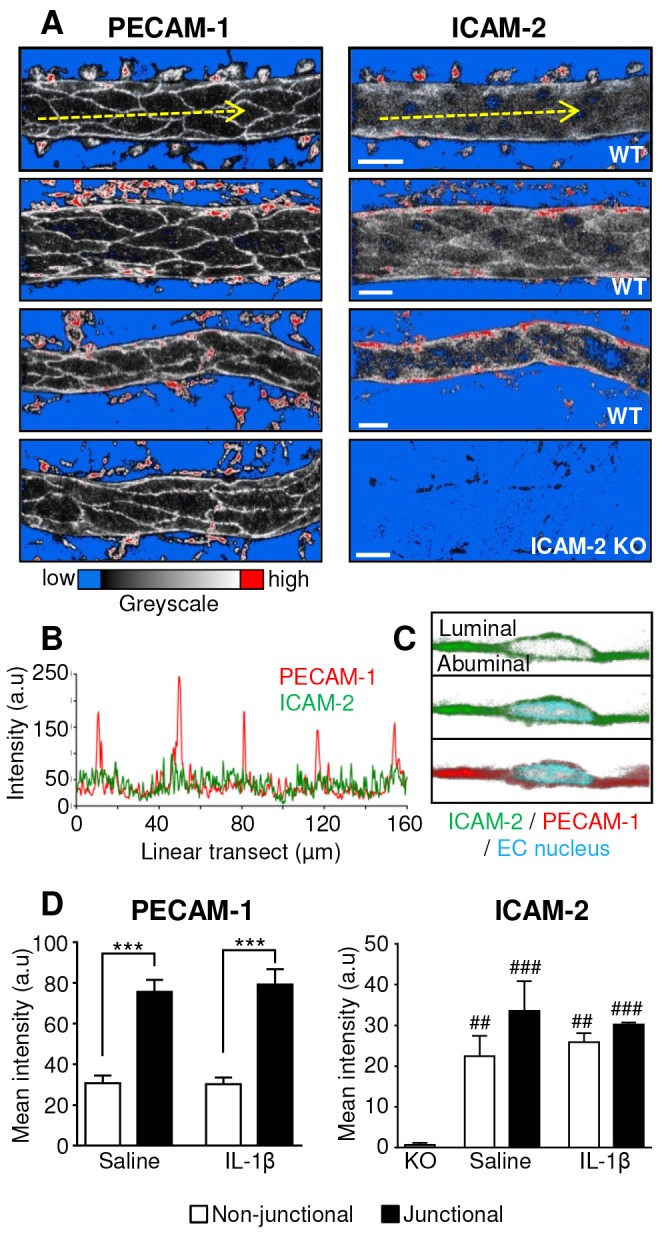
**PECAM-1 and ICAM-2 expression in cremasteric post-capillary venules.** Venular expression and localisation of PECAM-1 and ICAM-2 was investigated in saline and IL-1β-stimulated WT or ICAM-2 KO tissues by immunofluorescence labelling and post-acquisition analysis of junctional and non-junctional regions of endothelial cells. (A) Representative images of venules from WT tissues which were labelled *in vivo* with an i.s. injection of fluorescent anti-PECAM-1 or anti-ICAM-2 mAb. Anti-ICAM-2 mAb specificity was confirmed by immunolabelling cremaster muscles from ICAM-2 KO mice. The brightly labelled perivascular structures are phagocytic cells, which non-specifically take up antibodies during the *in vivo* labelling protocol. Scale bars: 20 µm. (B) Linear transect intensity profile of a two-dimensional maximal intensity projection of a PECAM-1- and ICAM-2-labelled vessel, showing high intensity PECAM-1 peaks at the EC junctions. (C) Draq5 was used to label the nuclei of ICAM-2- and PECAM-1-labelled vessels, and high-magnification optical sections were examined to determine whether PECAM-1 and ICAM-2 were expressed on the luminal and/or abluminal side of the nucleus, indicating expression on the luminal or abluminal EC surface. (D) Junctional and non-junctional distribution of PECAM-1 and ICAM-2 was quantified from 3D models of IL-1β- or saline-stimulated vessels using Imaris software (see supplementary material Fig. S1B for the isosurface method). *n* = 3–5 vessels per mice, three mice per group, error bars show s.e.m. ***P<0.001 between junctional or non-junctional regions; ^##^*P*<0.01, ^###^*P*<0.001 between WT and ICAM-2 KO (all Student's *t*-test).

Although ICAM-2 is commonly considered to be an endothelial cell junctional protein, we have previously shown that *in vivo* it is also expressed on the EC body (Woodfin et al., 2009). To extend these findings a detailed quantitative analysis of the expression of ICAM-2 in the mouse cremaster muscle microcirculation was performed. Specifically the level and localisation of ICAM-2 expression was investigated in comparison to other key EC junctional adhesion molecules, PECAM-1 and VE-cadherin, by immunofluorescence staining and confocal microscopy of fixed whole-mount cremaster muscles. ICAM-2, PECAM-1 and VE-cadherin were all expressed in cremaster muscle arterioles, venules and lymphatic vessels (supplementary material Fig. S1A). The expression profile of PECAM-1 and ICAM-2 in post-capillary venules, the primary sites of neutrophil TEM, was analysed in more detail. Whereas PECAM-1 showed enriched EC junctional expression, ICAM-2 was expressed to an almost equal level in both junctional and non-junctional regions of the endothelium (Fig. 1; supplementary material Fig. S1B). A similar expression profile was also found in ear dermal post-capillary venules (supplementary material Fig. S1C). High-magnification optical sections of vessels labelled for ICAM-2, PECAM-1 and Draq5 (a nuclear marker) showed that PECAM-1 and ICAM-2 were on the luminal side of the EC nuclei, confirming that the non-junctional ICAM-2 is expressed on the luminal surface of ECs (Fig. 1C). The specificity of our ICAM-2-labelling protocol was illustrated by lack of labelling of tissues from ICAM-2-deficient mice (Fig. 1). Of importance, the expression profile of ICAM-2, and also that of PECAM-1, was quantitatively similar in both saline and IL-1β-stimulated (50 ng, 4 hours) cremaster muscles (Fig. 1D). The presence of substantial amounts of constitutively expressed ICAM-2 on the EC body, as well as EC junctions, suggests that ICAM-2 might support luminal leukocyte–EC interactions in addition to mediating leukocyte TEM, a question that was investigated next.

### ICAM-2 supports dynamics of neutrophil luminal crawling

To enable investigation of the functional role of ICAM-2 in the dynamics of neutrophil–vessel interactions *in vivo*, ICAM-2 deficient mice were crossed with *LysM-EGFP-ki* mice yielding ICAM-2 knockout (KO) mice with endogenous expression of GFP in neutrophils and monocytes. Monocytes could be excluded from analysis by their substantially lower levels of GFP expression. Initial studies showed that as we have previously found with the pure ICAM-2 KO mice (Huang et al., 2006; Woodfin et al., 2009), the ICAM-2^−/−^/LysM-EGFP^+/−^ mice exhibit reduced IL-1β stimulated neutrophil extravasation but normal adhesion in cremasteric venules (supplementary material Fig. S2). To investigate dynamics of luminal neutrophil–vessel-wall interactions, neutrophil responses were analysed using confocal IVM as previously detailed (Woodfin et al., 2011). Briefly, the cremasteric vasculature of wild-type (WT), ICAM-2^−/−^/LysM-EGFP^+/−^, or LysM-EGFP^+/−^ mice treated with an anti-ICAM-2 blocking mAb or an isotype control [3 mg per kg of body weight, intravenously (i.v.)], were labelled with fluorescent anti-PECAM-1 mAb, and tissues were stimulated with IL-1β [50 ng, intrascrotal injection (i.s.)] for 2 hours. Following exteriorisation, selected post-capillary venules were imaged at 30-second intervals for a further 2 hours (∼30–40 minutes per vessel). Post-acquisition, the sequential image stacks were converted into dynamic 3D models, and the migratory behaviour of individual cells was manually tracked and quantified using the image analysis software Imaris. Examples of inflamed (IL-1β stimulated) and uninflamed (unstimulated) vessels, and the tracking of an individual neutrophil are shown in supplementary material Movies 1–3. No differences between WT and WT with isotype control treatments were seen, so these groups were pooled.

**Fig. 2.**
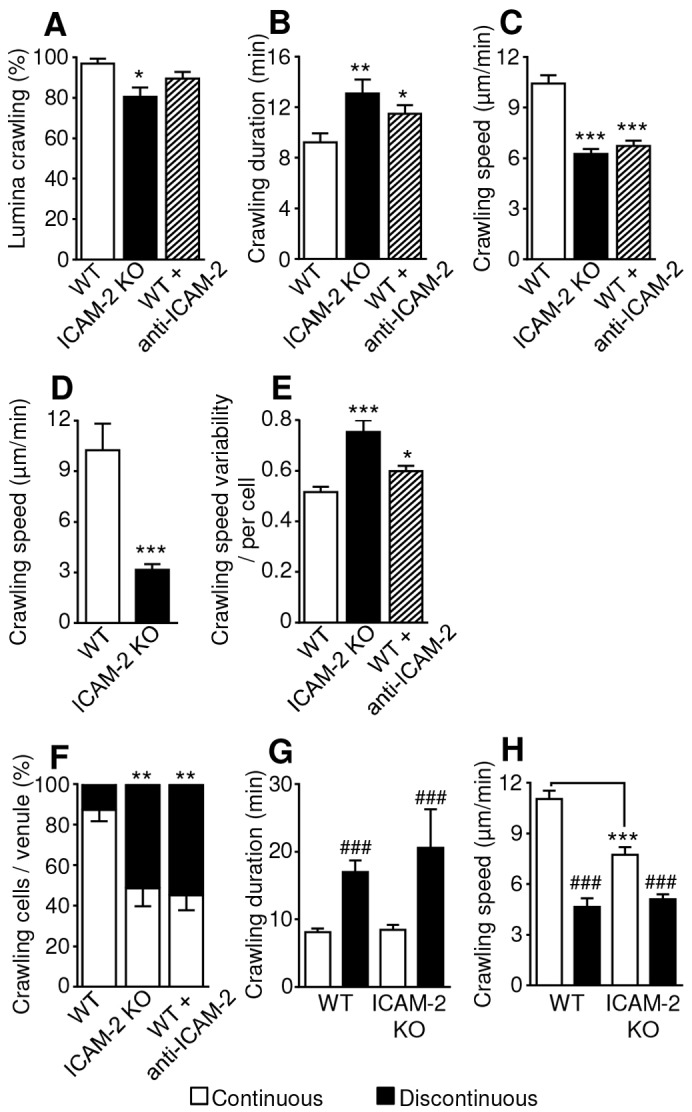
**Role of ICAM-2 in IL-1β stimulated neutrophil luminal crawling dynamics.** Cremasteric post-capillary venules of WT or ICAM-2^−/−^/LysM-EGFP^+/−^ mice, or WT LysM-EGFP^+/−^ mice treated with anti-ICAM-2 blocking mAb (3 mg per kg of body weight, i.v.) were analysed. Tissues were labelled *in vivo* with an i.s. injection of fluorescent anti-PECAM-1 mAb (4 µg i.s.) and co-injected with IL-1β (50 ng i.s.) 2 hours prior to exteriorisation. Sequential confocal images of selected venules were captured at 30-second intervals, for ∼30 minutes per venule, for a further 2 hours. (A) The behaviour of luminal neutrophils was analysed, and the percentage of adherent neutrophils that exhibited crawling per venule was quantified. Crawling dynamics were also quantified from these sequences. Mean (B) duration, (C) speed, (D) speed on EC bodies (and supplementary material Fig. S3), and (E) speed variability (standard deviation of crawling speed per cell, divided by the mean crawling speed per cell) of crawling cells is shown. (F) The behaviour of crawling cells was identified as being either continuous or discontinuous, and the percentage of these behaviours among all crawling cells was quantified per vessel. Other crawling dynamics of continuously and discontinuously crawling cells was compared, and the mean (G) duration and (H) speed of continuously and discontinuously crawling cells in each mouse strain is shown. *n* = 76–81 cells (from 10 vessels) in six or seven mice per group, error bars show s.e.m. *P<0.05, **P<0.01, ***P<0.001 between WT and antibody-treated or ICAM-2 KO mice; ^###^*P*<0.001 between continuous or discontinuous crawling parameters (Student's *t*-test or ANOVA).

Using the above technique, the frequency of crawling, and non-crawling or immobile cells within the luminally adherent population was quantified (supplementary material Movies 3 and 4). No difference in rolling frequency was seen in ICAM-2 KO animals (data not shown), and rolling cells were excluded from this analysis. In each group, the majority of adherent cells exhibited crawling behaviour (defined as movement of >5 µm during observation), with a small but significant reduction in crawling frequency in the ICAM-2 deficient animals [96.8±2.5% in WT and 80.4±4.7% in ICAM-2 KO (±s.e.m.)] (Fig. 2A). The observed duration of neutrophil crawling was significantly increased in ICAM-2 KO and antibody-treated mice [9.2±0.7 minutes in WT, 13.1±1.1 minutes in KO and 11.5±0.7 minutes in the anti-ICAM-2 mAb group (±s.e.m.)] (Fig. 2B). A significant reduction in mean crawling speed was also detected in ICAM-2 KO and WT mice treated with anti-ICAM-2 antibody [10.4±0.5 µm/minute in WT, 6.3±0.3 µm/minute in KO and 6.7±0.3 µm/minute WT mice treated with anti-ICAM-2 mAb (±s.e.m.)] (Fig. 2C). In order to determine whether ICAM-2 expressed on the luminal surface of ECs supports crawling or if EC junctional ICAM-2 plays the primary functional role, neutrophil crawling tracks were analysed in detail and time points during which crawling cells were only in contact with EC bodies, not junctions, were identified (supplementary material Fig. S3). This analysis showed a significant reduction in neutrophil crawling speed on EC bodies in the ICAM-2 KO vessels (Fig. 2D).

Of note, the variability of crawling speed of individual cells (standard deviation of crawling speed per cell, divided by the mean crawling speed per cell) was significantly higher in the ICAM-2 KO and antibody-treated mice (Fig. 2E). No difference was detected in neutrophil crawling straightness or displacement (data not shown). In each group, the net crawling displacement direction relative to blood flow was predominately with or perpendicular to the direction of blood flow, with few cells crawling against the direction of flow [87.2±6.5% in WT, 93.2±4.9% in ICAM-2 KO and 95.8±5.8% after anti-ICAM-2 mAb treatment (±s.e.m.)].

These data indicate that ICAM-2 is not essential for initial adhesion or the exhibition of crawling behaviour, but that there is a role for ICAM-2 in supporting efficient luminal crawling with respect to its velocity.

### ICAM-2 impacts on the profile of neutrophil crawling

While analysing crawling dynamics it was noted that neutrophils exhibited different modes of crawling. Specifically, some neutrophils exhibited continuous crawling, in that they were motile for the full duration of observations but others exhibited periods of immobility with a mean duration of ∼7 minutes. These behaviours have been termed ‘continuous’ and ‘discontinuous’ crawling respectively, and are illustrated in supplementary material Movies 5 and 6. Quantification of the frequency at which these responses occurred in IL-1β-stimulated tissues showed that whereas in WT mice the majority [87.2±5.6% (±s.e.m.)] of crawling cells had a continuous crawling profile, in ICAM-2 KO and animals treated with anti-ICAM-2 antibody there was a shift towards a greater frequency of discontinuous crawling (48.5±8.8% in KOs and 45.1±7.4% after anti-ICAM-2 treatment) (Fig. 2F), with a >50% reduction in continuous crawling. Not surprisingly, in both WT and ICAM-2 KO mice, neutrophils that exhibited discontinuous crawling also showed a significantly reduced average crawling speed and increased duration of luminal crawling (Fig. 2G,H), and the same effects were seen in the presence of anti-ICAM-2 mAb (data not shown). However, when only continuously crawling cells were compared between WT and ICAM-2 KO mice, the latter still showed a reduction in speed (Fig. 2H), collectively suggesting that in the absence of functional ICAM-2 there is a reduction in both neutrophil crawling speed and continuity.

### ICAM-2-mediated crawling might be mediated through MAC-1 ligation

The neutrophil integrin MAC-1 is a known ICAM-2 ligand, and it was hypothesised that ICAM-2–MAC-1 interactions could mediate the observed role of ICAM-2 in neutrophil crawling. The possibility that any observed effect of blocking MAC-1 was related to inhibition of MAC-1–ICAM-1 interactions was also considered, as this integrin is also known to bind ICAM-1 (Phillipson et al., 2006; Smith et al., 1989; Sumagin et al., 2010). As expected ICAM-1 was upregulated in IL-1β-stimulated tissues in both WT and ICAM-2 KO mice, but no evidence was found for ICAM-2 KO specific changes in ICAM-1 distribution or an additional compensatory increase in ICAM-1 expression (supplementary material Fig. S4).

**Fig. 3.**
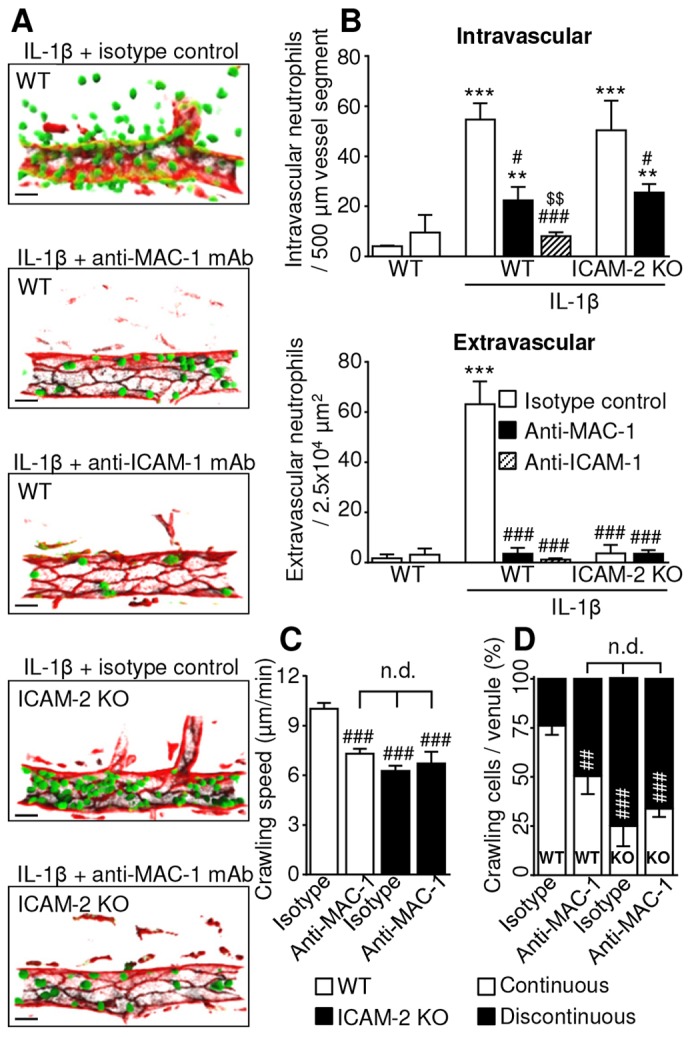
**MAC-1 interactions with ICAM-1 and ICAM-2 support luminal adhesion and crawling.** The contribution of ICAM-1 and ICAM-2 ligation of leukocyte MAC-1 to IL-1β-stimulated adhesion, crawling and extravasation was investigated. WT or ICAM-2^−/−^/LysM-EGFP^+/−^ mice were pre-treated with anti-ICAM-1 or MAC-1 blocking mAb, or isotype control (3 mg per kg of body weight, i.v.). Tissues were labelled *in vivo* with an i.s. injection of fluorescent anti-PECAM-1 mAb (4 µg i.s.), and co-injected with IL-1β (50 ng i.s.) 2 hours prior to exteriorisation. (A) The effect of genetic and pharmacological interventions was confirmed by capturing images at the end of the experiment (4 hours post stimulation) Scale bars: 10 µm. (B) The number of intravascular and extravasated neutrophils at this time point was quantified in each condition. Images and data are representative of three to five vessels per cremaster muscle (*n* = 3–8 mice per group). (C) Sequential confocal images of selected venules were captured at 30-second intervals for ∼30 minutes per venule, from 2–4 hours post stimulation. Neutrophil crawling speed was quantified in IL-1β-stimulated animals pre-treated with blocking mAbs or isotype control. (D) The percentage of continuously or discontinuously crawling neutrophils per venule was determined in each group. Data were obtained from analysis of 66–113 crawling cells from four to nine vessels in four to eight mice. Results are mean ± s.e.m. for all events analysed. **P<0.01, ***P<0.001 between unstimulated and IL-1β treated groups; ^#^*P*<0.05, ^###^*P*<0.001 between isotype and blocking antibody, or WT and KO, group; ^$^*P*<0.05, ^$$^*P*<0.01 between blocking antibodies (all ANOVA). n.d., not significantly different.

WT or ICAM-2 KO animals were given 3 mg per kg of body weight (i.v.) anti-MAC-1 or anti-ICAM-1 blocking mAb, or matched isotype control, and cremaster muscles were labelled for PECAM-1 and stimulated with IL-1β as described above. To confirm the effectiveness of these interventions, at the end of the live-imaging experiments, ∼4 hours post IL-1β, still images of post-capillary venules were captured and intravascular and extravasated neutrophil numbers were quantified (Fig. 3A,B). In the ICAM-2-deficient animals, extravasation but not luminal adhesion was reduced, and this was also seen when anti-ICAM-2 blocking mAb was used (not shown). By contrast, anti-ICAM-1 or anti-MAC-1 inhibition reduced both adhesion and transmigration, although ICAM-1 blockade has a more profound effect on adhesion than MAC-1 inhibition (Fig. 3A,B). The differential effect of blocking ICAM-1 or ICAM-2 on numbers of adherent neutrophils indicates that the roles of these proteins are not fully redundant in this model. These data, coupled with previously published work, suggest that ICAM-1–MAC-1 binding, along with other possible ICAM-1 ligands, such as LFA-1, supports luminal adhesion (Phillipson et al., 2006; Sumagin et al., 2010).

The role of MAC-1 and ICAM-2 interactions in luminal crawling was further investigated. Definitively excluding a contribution of ICAM-1–MAC-1 binding in these studies was not possible, as use of an ICAM-1 blocking antibody abolishes luminal adhesion and so precludes subsequent analysis of crawling dynamics. Owing to this limitation, the effect of an anti-MAC-1 blocking mAb in WT and ICAM-2 KO mice was compared on the basis that a lack of a cumulative effect of MAC-1 inhibition in ICAM-2 KO vessels would indicate that the observed effect represents the function of these two proteins interacting or acting in series within the same pathway.

Analysis of crawling speed showed that treatment of WT mice with anti-MAC-1 resulted in a reduction in crawling speed comparable to the reduction seen in ICAM-2 KO mice (Fig. 3C). When ICAM-2 KO mice were treated with anti-MAC-1 antibody, no further reduction in crawling speed was seen (10.2±0.34 µm/minute in WT, 7.3±0.3 µm/minute in WT with MAC-1 mAb, 6.3±0.3 µm/minute in ICAM-2 KO and 6.7±0.7 µm/minute in ICAM-2 KO with anti-MAC-1 mAb). The same lack of cumulative inhibition was seen when the percentage of continuously crawling cells was quantified in the presence of MAC-1 blockade (76.2±4.6% in WT with isotype, 50.4±9.1 in WT with anti-MAC-1 mAb, 25±10.2% in ICAM-2 KO with isotype control, and 33.8±4.1% in ICAM-2 KO with anti-MAC-1 mAb) (Fig. 3D).

**Fig. 4.**
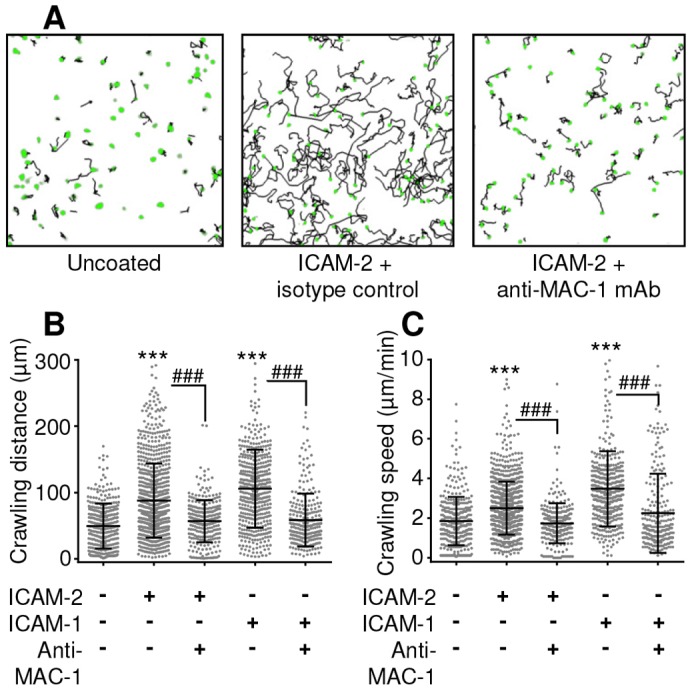
***In vitro* crawling on ICAM-1 or ICAM-2.** (A). Neutrophils were fluorescently labelled with Calcein-AM and seeded into un-coated (BSA blocked), or ICAM-2- or ICAM-1-coated chamber slides. Following adhesion, anti-MAC-1 mAb (5 µg/ml) was added to some groups. Images were captured at 2-minute intervals and crawling dynamics were analysed using Imaris. Examples show the position of neutrophils at *t* = 0 in green, and the crawling tracks over the following 40-minute period in black. Neutrophil crawling speed (B) and total crawling distance (C) was quantified on uncoated, ICAM-2- and ICAM-1-coated glass, with or without anti-MAC-1 mAb. Data were obtained from analysis of >300 crawling cells per group from four or five mice. The black lines show the mean±s.e.m. for all events analysed. ***P<0.001 between uncoated and ICAM-1/2 coated groups; ###*P*<0.001 between isotype and blocking antibody groups (all ANOVA).

In order to clarify the role of direct of ICAM-2–MAC-1 interactions in neutrophil crawling in the absence of any ICAM-1-mediated effects, and to exclude the possibility that another MAC-1 ligand might be acting in sequence with ICAM-2, we analysed crawling behaviour *in vitro*. Glass chamber slides were coated with BSA alone, BSA and ICAM-2, or BSA and ICAM-1. The crawling behaviour of freshly isolated and fluorescently labelled murine blood neutrophils on these surfaces was analysed in the presence or absence of anti-MAC-1 mAb (Fig. 4A; supplementary material Movies 7 and 8). A static system was used, as in the absence of ICAM-1-mediated firm adhesion very few cells crawling cells were retained under flow conditions with anti-MAC1 treatment. In these no-flow conditions, neutrophils were able to adhere to ICAM-2 and exhibited random crawling behaviour, albeit at a lower velocity than that seen *in vivo*. When anti-MAC-1 antibody was added to the chamber, there was a significant reduction in crawling speed and total distance crawled (Fig. 4B,C). A similar pattern of random crawling and anti-MAC-1 mAb inhibition of crawling speed and distance was seen on ICAM-1-coated surfaces and when CXCL1 was included in the ICAM-2 coating (not shown).

Collectively these *in vivo* and *in vitro* data provide evidence indicating that binding to ICAM-2 activates signalling pathways within neutrophils, and ICAM-2–MAC-1 binding supports the observed role of ICAM-2 in luminal neutrophil–EC interactions.

### ICAM-2 might facilitate the initiation of TEM

**Fig. 5.**
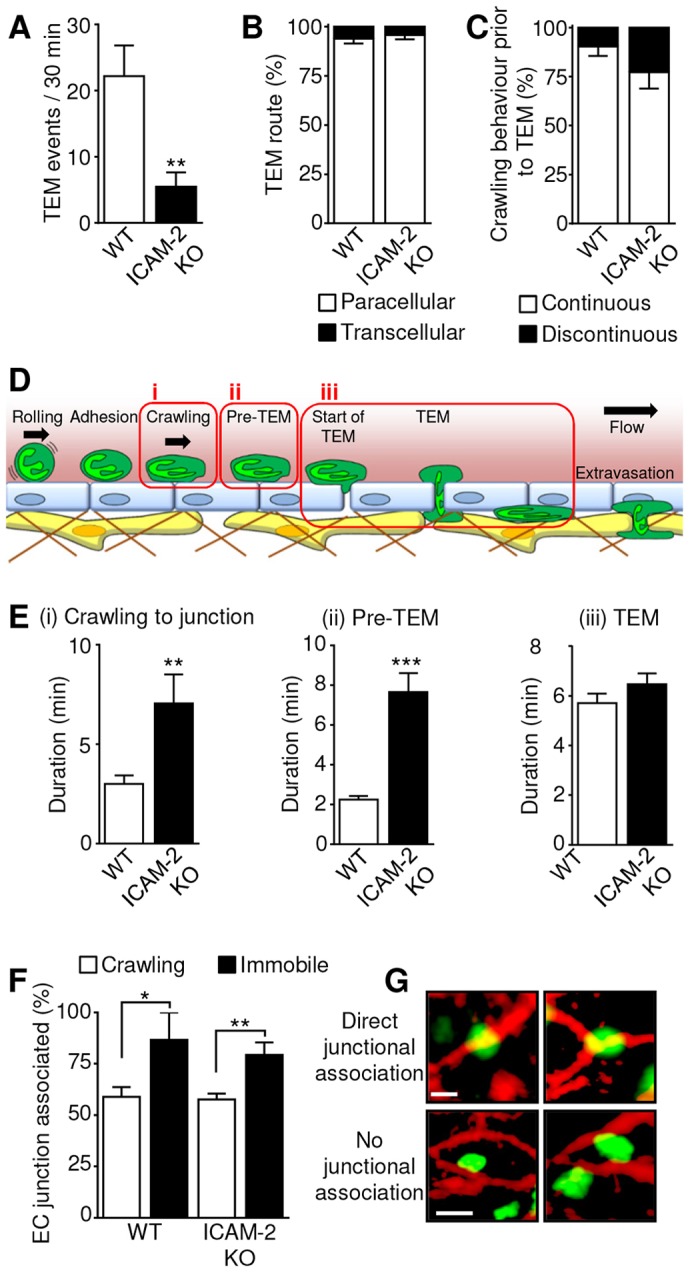
**IL-1β-stimulated TEM dynamics in ICAM-2 KO and WT mice.** The image sequences captured as described in Fig. 2 were also analysed for the occurrence of TEM. (A) The frequency at which TEM was observed following IL-1β stimulation was quantified in vessels from WT or ICAM-2^−/−^/LysM-EGFP^+/−^ mice over 30 minutes (the standard period of observation). (B) The route of TEM (paracellular or transcellular) was quantified as mean per venule. *n* = 60–196 cells from six to eight animals per group. (C) Crawling behaviour that preceded TEM (continuous or discontinuous) was quantified as mean per venule. *n* = 14–34 cells from six or seven animals per group. (D) The sequence of events from crawling through to completion of TEM was divided into three stages as illustrated. (i) Crawling: the period for which a neutrophil crawls within the lumen prior to reaching the site of TEM. (ii) Pre-TEM: the period for which a neutrophil interacts with the endothelial junction at the location of TEM prior to any visible disruption of the junctional integrity. (iii) TEM: the period from the first visible disruption of junctional PECAM-1 to the completion of migration to the sub-endothelial space. (E) The duration of stages i–iii as outlined in D was quantified. *n* = 14–34 cells (stage i), 23–76 cells (stage ii), or 20–99 cells (stage iii), from six or seven animals per group. (F) Periods of immobility were observed during luminal crawling (Fig. 2E), and the location of these events was analysed in relation to the location of EC junctions, and compared to the location of a random selection of normally crawling cells from the same vessels. *n* = 8 for WT and 77 for ICAM-2 KO immobile cells, and 250 randomly selected crawling cells from six or seven animals per group. (G) Examples of cells that were classified as being directly associated with EC junctions, and others that were only partially in contact with a junction, and not considered to be junction associated. Scale bars: 5 µm. Results are mean±s.e.m. **P<0.01, ***P<0.001 between WT and ICAM-2 KO (Student's *t*-test).

In addition to exhibiting crawling defects, ICAM-2 KO mice showed a significant reduction in the frequency of TEM (5.5±2.1 TEM events/30 minutes in KO mice and 22.18±4.6 events/30 minutes in WT) (Fig. 5A). In both groups, however, more than 90% of TEM events occurred through the paracellular route with no differences between the two mouse strains in terms of the route of TEM (Fig. 5B; supplementary material Movie 9).

A relatively small percentage of crawling cells analysed were observed to undergo TEM (17.4±5.9% in WT and 7.9±5.2% in ICAM-2 KO). This is partly owing to the technical limitations involved in tracking an individual neutrophil from initial adhesion to TEM within a limited time period and field of view. However, when a cell was observed to transmigrate, the crawling behaviour that preceded transmigration was examined. It was found that in both genotypes 80–90% of transmigrating neutrophils had previously crawled in a continuous rather than a discontinuous manner (Fig. 5C). Hence, in the ICAM-2 KO mice ∼50% of total crawling cells show discontinuous crawling, but >80% of those that transmigrated previously exhibited continuous crawling, indicating that those that do crawl continuously are much more likely to successfully transmigrate.

In order to ascertain how ICAM-2 was supporting neutrophil TEM, the cellular events leading to neutrophil TEM, and TEM itself were divided into distinct quantifiable steps (Fig. 5D), namely (i) crawling to the site of TEM, (ii) interaction with the junction prior to initiation of TEM, referred to as ‘pre-TEM’ (from arrival at the junction until the first visible disruption of the junctional PECAM-1), and (iii) TEM itself (the time from first disruption of the junction to completion of migration of the neutrophil through the junctional pore). In line with previous data (Fig. 2B), the observed duration of crawling to the site of TEM was significantly longer in ICAM-2 KO mice (3.3±0.4 minutes in WT and 7.1±1.5 minutes in ICAM-2 KO) (Fig. 5E, stage i). The interaction of neutrophils with their site of TEM prior to any detectable disruption of the PECAM-1-enriched junction (pre-TEM) was significantly prolonged in ICAM-2 KO mice in comparison to WT mice (3.2±0.3 minutes in KO and 1.1±0.1 minutes in WT) (Fig. 5E, stage ii). Interestingly no difference in the duration of TEM itself was noted, and in both WT and ICAM-2 KO mice the duration of this step was ∼6 minutes (Fig. 5E, stage iii). Collectively these results show that in the present IL-1β-driven acute inflammation ICAM-2 facilitates neutrophil crawling to EC junctions and the initiation of TEM but has no impact on the route or dynamics with which neutrophils breach EC junctions.

The location of the observed periods of immobility during discontinuous crawling in relation to EC junctions was analysed, and compared to the location of a random selection of normally crawling leukocytes in these vessels. The majority of these events (79%) within ICAM-2 KO vessels were directly associated with an endothelial junction, compared to only 56% of normally crawling cells (Fig. 5F,G). In WT vessels, periods of immobility were also predominantly associated with EC junctions, but were much less frequently seen, and only eight periods of immobility were observed in WT tissues. Coupled with the data showing that there was a prolonged interaction with the junction prior to initiating TEM (Fig. 5E, stage ii), these findings might indicate that the periods of immobility during crawling represent unsuccessful attempts to initiate junctional opening, suggesting a possible role for ICAM-2 in the early stages of paracellular TEM.

## DISCUSSION

The current study employed a recently developed confocal imaging system to investigate the role of ICAM-2 in the dynamics of luminal neutrophil–venular-wall interactions *in vivo*. The results revealed previously unreported roles for luminal ICAM-2 in supporting efficient crawling through ligation of neutrophil MAC-1. Furthermore, the current data suggest that ICAM-2 might also be important in the initiation or early stages of paracellular TEM. Collectively these roles facilitate IL-1β-stimulated extravasation of neutrophils and might thus be involved in supporting an efficient innate immune response to infection and injury.

Unlike the expression of other EC adhesion molecules (e.g. ICAM-1 and VCAM-1) that have been widely studied *in vitro* and *in vivo* (Vestweber, 2007), the expression profile of ICAM-2 has not been extensively analysed. To address this issue, we extended our previous work reporting on the heterogeneous distribution of ICAM-2 on the non-junctional surface of cremaster venular ECs (Woodfin et al., 2009) to a detailed quantitative analysis of EC junctional and non-junctional expression of ICAM-2 as compared to the EC junctional protein PECAM-1. In the mouse cremaster muscle ICAM-2 was detected at non-junctional and junctional regions of ECs to a comparable level, in contrast to PECAM-1 expression, which was largely detected at EC junctions. This expression profile was also seen in mouse ear dermal venules, suggesting that functional implications obtained from studies within the cremaster might be broadly applicable to other tissues. ICAM-2 has generally been considered to be a constitutively expressed EC junctional protein (de Fougerolles et al., 1991; Vestweber, 2007), and no regulation of the intensity or distribution of either of these proteins was seen following IL-1β stimulation. However, the constitutive luminal expression of ICAM-2 reported in the present study opens up novel functional possibilities. A limited number of studies have reported altered expression of ICAM-2, although these include upregulation in clinical scenarios of a more chronic and complex nature, such as lymphoid malignancies (Renkonen et al., 1992) or Crohn's disease (Bernstein et al., 1998), or downregulation through inhibition of transcription following prolonged exposure to cytokines in HUVECs (McLaughlin et al., 1998). Of note, both ICAM-2 KOs and WT mice upregulated ICAM-1 expression following IL-1β stimulation, and no differences in the upregulation or distribution of ICAM-1 was seen in these animals.

Previous work from our group using fixed tissues has identified sequential and non-redundant roles for ICAM-2, JAM-A and PECAM-1 during paracellular neutrophil transmigration (Woodfin et al., 2009), with a role for ICAM-2 being identified in the earliest stages of TEM. In the light of the current findings showing high luminal expression of ICAM-2, we hypothesised that ICAM-2 might play a role in regulating luminal leukocyte–endothelial interactions, in addition to a possible role during TEM itself. This was investigated through the application of a high-resolution confocal IVM system (Proebstl et al., 2012; Woodfin et al., 2011) to analysis of leukocyte–EC interactions within cremasteric venules of WT or ICAM-2-deficient *LysM-EGFP-ki* mice, which have GFP-positive neutrophils. These studies showed that in WT animals most luminally adherent neutrophils exhibited some crawling motility and that overall this was not dramatically altered in ICAM-2 KO mice. Similar results were obtained in WT mice treated with a blocking anti-ICAM-2 mAb, findings that are largely in agreement with previously published work (Phillipson et al., 2006). However, in ICAM-2 KO mice or mice treated with anti-ICAM-2 mAb the mean crawling velocity of neutrophils was significantly slower and there was greater variability in the speed of individual crawling tracks. An increase in the observed duration of crawling was also noted, indicating prolonged luminal interactions prior to either TEM, or disengagement from the endothelium and return to the circulation. Furthermore, in the absence of functional ICAM-2, crawling neutrophils frequently exhibited a discontinuous crawling behaviour characterised by prolonged periods of immobility. So although ICAM-2 is not essential for luminal crawling to occur, possibly because ICAM-1 has some contribution to this response (Phillipson et al., 2006), our data demonstrate for the first time a role for ICAM-2 in mediating the speed and continuity of neutrophil luminal motility.

In addition to EC expression, neutrophils also express ICAM-2 and the potential role of the latter in the observed findings cannot be ruled out. Indeed neutrophilic expression of ICAM-2 has recently been linked to stabilisation of neutrophil rolling (Sundd et al., 2012). However, through the use of passive transfer experiments, we have previously obtained evidence for the involvement of EC ICAM-2 and not leukocyte ICAM-2 in leukocyte extravasation (Woodfin et al., 2009), strongly suggesting a non-redundant role for EC ICAM-2 in leukocyte–EC interactions.

To investigate the mechanism through which ICAM-2 mediates neutrophil crawling dynamics *in vivo*, the role of the leukocyte integrin MAC-1, a known binding partner for ICAM-2 (Xie et al., 1995), was investigated. Of relevance, MAC-1 has previously been implicated in leukocyte motility and crawling (Phillipson et al., 2006; Sumagin et al., 2010). In line with previous findings (Huang et al., 2006; Woodfin et al., 2009), normal levels of luminal neutrophil adhesion, but reduced neutrophil extravasation, were seen in ICAM-2-deficient conditions. In contrast, anti-MAC-1 mAb treatment partially reduced adhesion in both WT and ICAM-2 KO mice, and substantially inhibited extravasation in WT animals. The contribution of ICAM-1, which has overlapping ligand specificities with ICAM-2 (Ding et al., 1999; Li et al., 1993; Staunton et al., 1989; Xie et al., 1995), was also investigated. Although ICAM-2 blockade or genetic deletion had no effect on adhesion, ICAM-1 blockade had a profound inhibitory effect on neutrophil adhesion as a result of which neutrophil extravasation was almost totally abolished.

In addition to this ICAM-2 independent effect on adhesion, MAC-1 inhibition also led to a reduced mean crawling velocity of the remaining neutrophils and an increased frequency of disrupted crawling events. These findings show a similar profile of luminal leukocyte dynamics to those noted in ICAM-2 KO mice, and dual inhibition of both MAC-1 and ICAM-2 had no cumulative effects on luminal dynamics, suggesting that interaction of these molecules might support efficient neutrophil crawling in the present *in vivo* model.

In order to more directly investigate the role of MAC-1 in ICAM-2 mediated crawling we employed a model in which the functional roles of ICAM-2 or ICAM-1 in neutrophil crawling could be examined in isolation. These results confirmed that MAC-1 blockade inhibits crawling on ICAM-2 in the absence of other MAC-1 ligands, supporting the assertion that ICAM-2–MAC-1 ligation, as opposed to sequential functions within a pathway, can support neutrophil crawling. In this simple *in vitro* model, crawling on ICAM-1 is also inhibited by anti-MAC-1 mAb, and the functions of ICAM-1 and ICAM-2 appear to be redundant. However the *in vivo* data presented in this and other studies demonstrates that ICAM-1 cannot fully compensate for a lack of ICAM-2 within post-capillary venules. The similarity in crawling behaviour on ICAM-1 or ICAM-2 *in vitro* might indicate that the different roles for these proteins observed *in vivo* are not only dependent on the leukocyte locomotive machinery. Whether the functional differences *in vivo* are due to differences in EC expression levels, the binding specificity or the affinity of these endothelial adhesion molecules to different integrins (Casasnovas et al., 1997; Li et al., 1993; Li et al., 1995; Staunton et al., 1989; Xie et al., 1995), the potential unique roles for ICAM-2 in the opening of or entry to EC junctions, or a combination of these possibilities, is not known at this time.

Collectively these findings, along with previously published studies (Phillipson et al., 2006; Smith et al., 1989; Sumagin et al., 2010), suggest that both ICAM-1–LFA-1 and ICAM-1–MAC-1 pathways support initial neutrophil adhesion, whereas ICAM-2–MAC-1 interaction supports efficient luminal crawling post-adhesion, possibly through selective activation at the leading edge of crawling neutrophils (Hidalgo et al., 2009).

In addition to a role in neutrophil luminal crawling dynamics, we also investigated how TEM frequency and dynamics were affected by the absence of ICAM-2. Whereas ICAM-2 KO mice supported comparable numbers of adherent and crawling neutrophils, very few cells exhibited a successful TEM response. Of note, the TEM responses that did occur in both WT and KO mice were primarily through the paracellular route, in line with our previous findings (Woodfin et al., 2011). In the light of the above findings showing a role for ICAM-2 in efficient neutrophil crawling, we hypothesised that disrupted crawling dynamics in ICAM-2 KO mice might account for reduced neutrophil TEM events. In support of this, detailed analysis of crawling behaviour of cells prior to successful TEM in the ICAM-2 KO mice indicated a greater frequency of ‘continuous crawling’ than is seen among the total crawling cell population. These observations suggest that slower, discontinuous and prolonged crawling hinders neutrophils in initiating a TEM response. In addition, the duration of neutrophil contact with EC junctions at sites of TEM prior to any observable disruption of the junction was extended. However, although collectively we observed delays in migration to, and opening of, EC junctional pores, once TEM had commenced, the timeframe of breaching of EC junctions was similar in both WT and ICAM-2 KO mice.

These findings are in line with our previous *ex vivo* studies that showed that neutrophils arrest at an early stage of TEM in ICAM-2 KO mice (Woodfin et al., 2009), but significantly extend these findings in terms of the observation of the neutrophil behaviour prior to TEM, that is, the frequency at which these junction-associated neutrophils progress to TEM, remain stationary or move away from the junction. In addition, we have been able to demonstrate that TEM progresses normally once initiated, without further defects or delays in traversing the EC junction. This striking absence of any role for ICAM-2 in the migration dynamics once the neutrophil has entered the paracellular junction suggests that the independent and sequential functions of other EC junctional proteins in TEM come into play during the later stages of migration through the junction (Bixel et al., 2010; Schenkel et al., 2002; Sullivan et al., 2013; Woodfin et al., 2009; Woodfin et al., 2011).

The location of periods of immobility, which were frequently exhibited by crawling neutrophils in the ICAM-2 KO mice, were strongly associated with the EC junctions, and this observation could provide a link between the defects in crawling continuity, prolonged duration of pre-TEM junctional interactions, and the overall reduced frequency of TEM. Putting these results together a possible model to explain our findings is that in the absence of EC ICAM-2 the greater frequency of transiently immobile neutrophils represents cells that are attempting to enter the EC junctions, and when unable to do so they resume crawling.

The prolonged interactions with EC junctions observed prior to TEM (pre-TEM) described above, and the periods of immobility associated with EC junctions which do not progress to TEM both seem to involve neutrophil MAC-1, but whether the observed dynamics arise from a defective mechanism within the neutrophils or the endothelium is unknown. The lack of ICAM-2 ligation might reduce the ability of neutrophils to form protrusions into the EC junction, or simply that less-efficiently crawling neutrophils become stuck at EC junctions that are not permissive for TEM, rather than continuing to crawl efficiently until a more suitable site for TEM is identified. Alternatively, ICAM-2 ligation at EC junctional contacts might trigger signalling events within ECs through cytoskeletal proteins such as α-actinin and ezrin-radixin-moesin proteins, which facilitate the opening of EC junctions and allow the junction-associated neutrophils to begin TEM (Heiska et al., 1996; Heiska et al., 1998; Helander et al., 1996; Yonemura et al., 1998).

In conclusion, the results presented here demonstrate a number of novel roles for ICAM-2 in neutrophil–EC interactions that support efficient extravasation, namely supporting efficient neutrophil crawling dynamics and in the opening or identification of EC junctions. It remains to be determined whether these responses are inter-related or represent distinct roles for ICAM-2, but speculating that crawling is supported by activation of leukocyte locomotive machinery (e.g. through ligation of MAC-1), whereas EC junctional opening is be facilitated by the formation of neutrophil protrusions or signalling within ECs provides an attractive platform for future investigations. 

## MATERIALS AND METHODS

### Animals

Male wild-type (WT) C57BL/6 mice (20–25 g) were purchased from Charles River Laboratories (Margate, UK). ICAM-2 KO mice (Gerwin et al., 1999) were a gift from Britta Engelhardt (Theodor Kocher Institute, University of Bern, Switzerland). The primary strain used for live imaging were mice in which the gene for EGFP has been knocked-in to the lysozyme M (LysM) locus (*LysM-EGFP-ki*) (Faust et al., 2000), resulting in the expression of EGFP in myelomonocytic cells, with high levels of EGFP in neutrophils and to a lesser extent monocytes. ICAM-2^−/−^/LysM-EGFP^+/−^ mice were generated in-house by interbreeding ICAM-2 KO mice with *LysM-EGFP-ki* mice, yielding animals deficient in ICAM-2 protein with GFP-positive neutrophils and monocytes. Only mice heterozygotic for *LysM-EGFP-ki* were used in the study. All genetically modified animals were on a C57BL6 background. All animals were housed in individually ventilated cages, and facilities were regularly monitored for health status and infections. All experiments were performed under the UK legislation for the protection of animals, and at the end of all *in vivo* procedures animals were humanely killed by cervical dislocation in accordance with UK Home Office regulations. 

### Reagents

Recombinant mouse interleukin-1β (IL-1β), recombinant mouse ICAM-2 and recombinant mouse and human ICAM-1 were purchased from R&D systems (Abingdon, UK). Tyrode's salts was purchased from Sigma-Aldrich (Poole, YK). R10 tissue culture medium was from Gibco (NY, USA). Anti-ICAM-2 monoclonal antibody (mAb) (3C4; rat IgG2a) and isotype control mAbs (rat IgG2a and rat IgG2b) were obtained from Serotec (Kidlington, UK). Anti-ICAM-1 mAb (YN-1; rat IgG2b) and anti-PECAM-1 mAb (C390; rat IgG2a) were from eBiosciences (Hatfield, UK). Anti-MAC-1 mAb (M1/70; rat IgG2b), Alexa-Fluor-488-labelled anti-ICAM-2 mAb (3C4; rat IgG2a) was purchased from Biolegend (Cambridge, UK). Monoclonal antibody Alexa Fluor conjugation kits and Calcein-AM were from Invitrogen (Paisley, UK), and the anti-PECAM-1, anti-VE-cadherin and anti-ICAM-1 mAbs were conjugated to either Alexa Fluor 488, 555 or 647 in-house. 

### Analysis of EC protein expression

Confocal microscopy was used to analyse endothelial expression of PECAM-1, VE-cadherin, ICAM-2 and ICAM-1 in saline (control) or IL-1β-stimulated cremaster muscles or ear dermis from WT and ICAM-2 KO mice. Animals were sedated through intramuscular (i.m.) injection of 1 ml/kg anaesthetic (40 mg per kg of body weight ketamine and 2 mg per kg of body weight xylazine in saline), and intrascrotal (i.s.) injection of IL-1β (50 ng/400 µl saline) or saline alone. Saline or IL-1β were co-administered with non-blocking Alexa-Fluor-conjugated anti-PECAM-1 (2 µg), anti-ICAM-1 (4 µg) or anti-ICAM-2 (4 µg) mAbs. For VE-cadherin labelling, tissues were fixed with 4% PFA, blocked and permeabilised, then incubated with Alexa-Fluor-conjugated anti-VE-cadherin. For analysis of the mouse ear dermis, anti-ICAM-2 mAb was given (i.v. 3 mg per kg of body weight) prior to cervical dislocation, and ears were removed and fixed in 4% PFA. The dermis was separated from the cartilage layer, blocked and permeablised, incubated with a fluorescent secondary antibody to anti-ICAM-2 mAb (1 µg), then with Alexa-Fluor-conjugated anti-PECAM-1 mAb (2 µg).

Tissues were viewed using a Leica SP5 confocal microscope incorporating a 20× water-dipping objective (NA 1.0). *z*-stack images of three to five post-capillary venules per cremaster with 20–45 µm diameter were selected for analysis (three or four mice per group). Image processing and quantification of protein expression levels on venular ECs was carried out using Imaris (Bitplane) 3D analysis software. A differential analysis of the intensity of EC junctional and non-junctional expression levels was carried out by creating isosurfaces (Proebstl et al., 2012) with intensity threshold settings on either the high-intensity PECAM-1 at the EC junctions, or on the low-intensity PECAM-1 on the EC bodies. The intensity of PECAM-1, ICAM-1 and ICAM-2 within these regions could then be determined in saline and IL-1β-stimulated cremaster muscles (supplementary material Fig. S4). The linear intensity profile of protein expression (Fig. 1B) was quantified from a maximal intensity projection of the *z*-stack using the Leica LAS-AF software.

### Luminal leukocyte–EC interactions *in vivo*

Luminal leukocyte–EC interactions were analysed using confocal IVM as previously described (Proebstl et al., 2012; Woodfin et al., 2011). The cremaster muscles of sedated WT or ICAM-2^−/−^/LysM-EGFP^+/−^ mice were stimulated with i.s. saline or IL-1β, co-administered with a non-blocking Alexa-Fluor-555-labelled PECAM-1 antibody (2 µg) to visualize the endothelium. For ICAM-2, ICAM-1, and MAC-1 blocking experiments, the blocking or isotype control mAbs were administered i.v. (3 mg per kg of body weight) 15 minutes before IL-1β stimulation.

Mice were prepared for confocal IVM 2 hours after i.s. injections in order to observe firmly adherent leukocytes undergoing crawling and/or TEM for a further 2 hours. Briefly, mice were anesthetized by intraperitoneal (i.p.) injection of ketamine (100 mg per kg of body weight) and xylazine (10 mg per kg of body weight) and maintained at 37°C on a custom-built, heated microscope stage. The cremaster muscle was exteriorized, pinned out flat over the optical window of the stage, and superfused with warm Tyrode's balanced salts solution.

Images were acquired by sequential scanning of the 488 and 561 nm channels at a resolution of 1024×350 pixels in the *x*,*y* plane and 1.0 µm steps in the *z* plane, corresponding to a voxel size of 0.41×0.41×0.99 µm in the *x*,*y*,*z* planes, respectively. Confocal images were acquired with a Leica SP5 confocal microscope using a resonance scanner of 8000 Hz. Acquisition of a single *z*-stack of ∼60 images routinely took ∼30 seconds. Images were captured every 30 seconds for a further 2 hours, for 30–40 minutes per vessel [see movies for examples of an uninflamed vessel (supplementary material Movie 1) and an IL-1β-stimulated inflamed vessel (supplementary material Movie 2)].

To confirm the effect of genetic or pharmacological interventions, still images of three to five post-capillary venules per cremaster muscle were taken at the end of the experiment (∼4 hours after i.s. injections), and intravascular and extravascular leukocytes were quantified for a 500-µm vessel length for intravascular, and a 500-µm vessel length extending 50 µm into the perivascular tissue for extravasated cells (2.5×10^4^ µm^2^).

### Analysis of luminal leukocyte crawling dynamics

Post-acquisition sequential *z*-stacks were analysed using the software Imaris, which renders the stacks of optical sections into sequential 3D models, thereby enabling the dynamic interactions between luminal EC and leukocytes to be observed, tracked and analysed. All images and videos show half vessels to enable clear visualization of the luminal surface of the vessel wall. Using this technique, luminal leukocyte crawling and TEM profile and dynamics were quantified.

Specific parameters were established for investigating the profile and dynamics of leukocyte luminal crawling on ECs. For each image sequence, luminal cells that were clearly visible in terms of their location and dynamics for five or more frames (>2.5 minutes), were manually tracked and analysed. Approximately 70 crawling cells were analysed from three to eight mice per group. Crawling leukocytes were defined as any leukocyte which had a displacement more than approximately half a cell radius (5 µm) over the period of observation (supplementary material Movie 3). Non-crawling, or stationary, cells were defined as any leukocyte which had a displacement less than half a cell radius (∼5 µm) over the period of observation (supplementary material Movie 4).

Individual luminal crawling paths were tracked manually using Imaris, and the following parameters were quantified: duration of crawling (minutes), speed of crawling (µm/minute), distance crawled (µm), total displacement (straight line distance between the first and last track point), crawling displacement direction relative to blood flow, crawling straightness (ratio of crawling displacement length to crawling track length) and crawling speed variation (standard deviation of the crawling speed per cell, divided by the mean crawling speed per cell).

Two distinct types of leukocyte crawling behaviours were routinely observed, ‘continuous crawling’ and ‘discontinuous crawling’, and the frequency at which they occurred was quantified. Leukocytes were classified as exhibiting continuous crawling if they were mobile for the duration of observation. Supplementary material Movie 5 shows an example of a continuously crawling cell. Discontinuous crawling was defined as crawling behaviour with periods of immobility of 2.5 minutes or greater during the period of observation. Immobility was defined by a cell displacement of less than approximately half a cell radius (5 µm). See supplementary material Movie 6 for an example of discontinuous crawling.

The location of each period of immobility exhibited by crawling cells was quantified in relation to EC junctions. Neutrophils were determined to be directly associated with EC junctions or as partially and not in contact with EC junctions. Cells with partial contact with an EC junction were not deemed to be junction associated, as the morphology of the ECs and their junctions mean that any randomly placed neutrophil-sized object would often have some degree of junctional contact, and this is not equivalent to the position cells are seen in immediately prior to commencing TEM (see Fig. 4G for examples). The location of periods of immobility were compared to the location of randomly selected crawling cells from the same vessels, taken from the first and last time point of each image sequence.

### Analysis of leukocyte TEM dynamics

TEM events which were clearly visible for the duration of their migration through the EC layer were analyzed. Paracellular and transcellular TEM events were identified through the observation of leukocyte TEM through the ECs, and the occurrence of transient pores in EC junctional or non-junctional PECAM-1 staining during a TEM event (Woodfin et al., 2011). Luminal events leading up to TEM were also analysed. This included the duration of luminal crawling to the EC junction that would be the eventual site of TEM, and the duration for which leukocytes were in contact with the EC junction prior to commencing TEM (the point at which junctional disruption is observed), which was termed ‘pre-TEM’.

It is important to note that both crawling and TEM analyses relate only to the time for which each cell was observed during the limited 30–40-minute observation period, and within the ∼200-µm-long vessel segment in the field of view. In many cases, cells that were already adherent at the beginning of observation, were still exhibiting luminal crawling at the end of the period of observation, or simply crawled out of the field of view, and as such their ultimate fate is unknown. It is only possible to observe the full sequence of initial adhesion, crawling and TEM in a small subset of cases. The limitations inherent here are, however, equal across all groups, so recorded differences in behaviour between groups are valid in terms of relative difference.

### *In vitro* crawling assay

Neutrophils were isolated from the blood of male C57BL/6 mice using an anti-Ly6G MicroBead kit (Miltenyl Biosciences) as per the manufacturer's instructions. Neutrophil purity obtained using these kits is >90% (not shown). The neutrophils were fluorescently labelled with Calcein-AM (1 µg/ml, 30 minutes at 37°C), and 50,000 neutrophils per chamber were seeded into culture slides (BD Flacon) and allowed to adhere. Culture slides were uncoated, or coated with recombinant human ICAM-1 (2.5 µg/ml) or recombinant mouse ICAM-2-FC (10 µg/ml) overnight, then blocked with 10% BSA for 2 hours. Isotype control or anti-MAC-1 mAbs were added (10 µg/ml) following neutrophil adhesion and removal of non-adherent cells. Adherent neutrophils were imaged at 2 min intervals for 40 minutes using an Olympus IX8 inverted microscope with a 10× objective lens, while being maintained at 37°C and 10% CO_2_. Crawling dynamics were quantified using Imaris, and any remaining non-adherent cells were excluded from analysis.

### Statistics

Results are presented as mean±s.e.m. Statistical significance was assessed by Student's *t*-test or one-way analysis of variance (ANOVA) with the Newman-Keuls multiple comparison test. *P*-values below 0.05 were considered significant.

## Supplementary Material

Supplementary Material
