# Supplementary material for: ICAM-2 facilitates luminal interactions between neutrophils and endothelial cells in vivo
Source: J Cell Sci. 2014 Feb 1;127(3):620–9. doi: 10.1242/jcs.137463 (PMC4007766; doi:10.1242/jcs.137463)
Supplement: Supplementary Material [file supp_127_3_620__index.html]

ICAM-2 facilitates luminal interactions between neutrophils and endothelial cells in vivo — Supplementary Material 

# ICAM-2 facilitates luminal interactions between neutrophils and endothelial cells *in vivo*

## JCS137463 Supplementary Material

**Files in this Data Supplement:**

- **Supplementary Material PDF**
