# Supplementary material for: ICAM-2 facilitates luminal interactions between neutrophils and endothelial cells in vivo
Source: J Cell Sci. 2014 Feb 1;127(3):620–9. doi: 10.1242/jcs.137463 (PMC4007766; doi:10.1242/jcs.137463)
Supplement: Supplementary Material [file supp_127.3.620_JCS137463.pdf]

Supplementary Figures

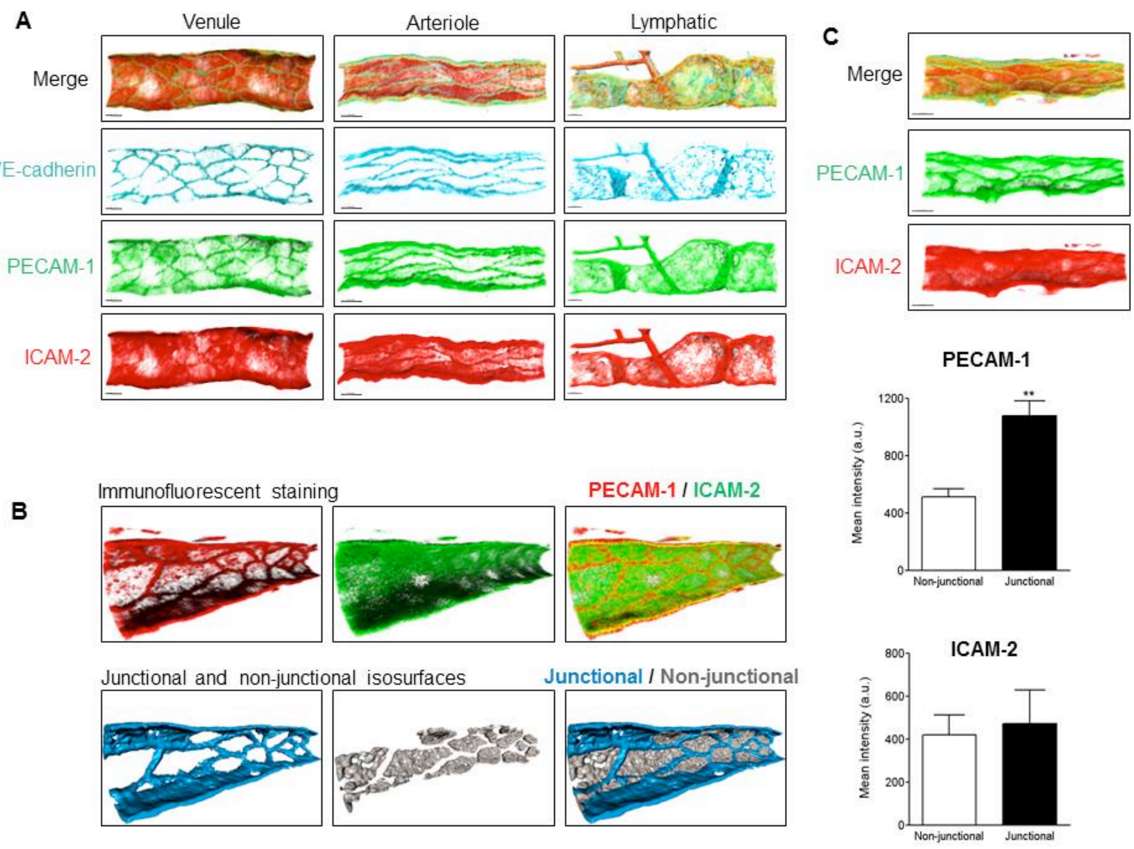

Fig S1. VE-cadherin, PECAM-1 and ICAM-2 expression in cremasteric and dermal vasculature.

(**A**) VE-cadherin, PECAM-1 and ICAM-2 expression on cremasteric venules, arterioles and lymphatic vessels. Tissues were labelled *in vivo* for PECAM-1 (green) and ICAM-2 (red), and post fixation for VE-cadherin (blue) using Alexafluor-488, -555 or -647 tagged mAbs. (**B**) Example showing construction of isosurfaces using intensity thresholding on PECAM-1 high junctions, and PECAM-1 low cell bodies in post capillary venules. Intensity of PECAM-1 (red/blue) and ICAM-2 (green/grey) within these isosurfaces could then be quantified. Venular expression and localisation of PECAM-1 and ICAM-2 in the mouse ear dermis. (**C**) Junctional and non-junctional intensity of PECAM-1 and ICAM-2 were quantified in venules from the mouse ear dermis. Scale bars represents 20 µm. N = 3 vessels per mice, 3 mice per group, error bars show s.e.m. Statistically significant (T-test) differences between junctional and non-junctional regions are indicated by asterisks, **P<0.01.

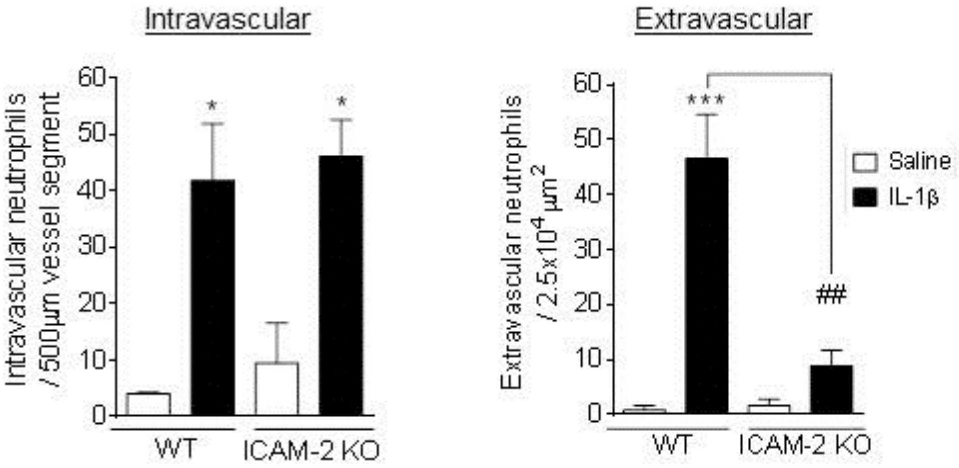

Fig S2. Role of ICAM-2 in IL-1β-induced neutrophil extravasation.
Cremasteric post-capillary venules of WT or ICAM-2$^{-/-}$/LysM-EGFP$^{+/-}$ mice were analysed. Tissues were labelled *in vivo* with an i.s. injection of fluorescent anti-PECAM-1 mAb (4 ng), co-injected with IL-1β (50 ng). Quantification of intravascular (left panel) and extravascular (right panel) neutrophils in WT or ICAM-2$^{-/-}$/LysM-EGFP$^{+/-}$ mice after saline or IL-1β stimulation (50ng i.s., 4 hours). N = 3-5 vessels per mice, 3-7 mice per group, error bars show s.e.m. Statistically significant (ANOVA) differences between IL-1β or saline stimulated tissues are indicated by asterisks, *P<0.05 and ***P<0.001. Differences between WT and KO responses are indicated with hash symbols, ##P<0.01.

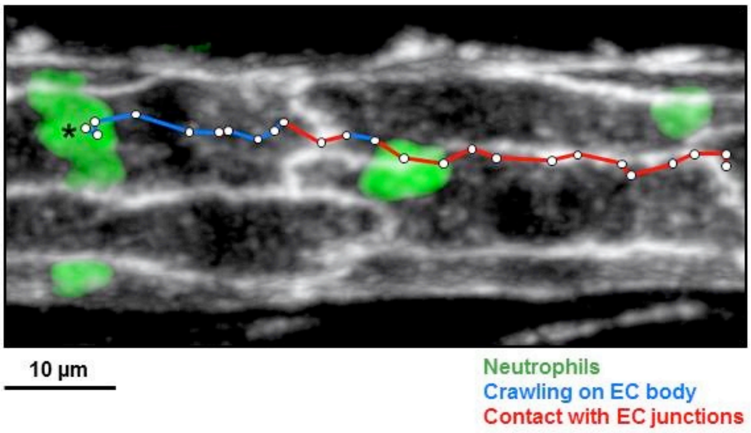

Fig S3. Crawling on EC junctions and cell bodies.
Example of a neutrophil crawling track, with the cell of interest indicated with an asterisk and shown at T=0. The movement of this cell during each subsequent 30 second time point (for a total of 31 min in this example) was classified as following a non-junctional (blue) route, or having some degree of junctional contact (red). The speed (µm/min) of each non-junctional time point was recorded, and the non-junctional crawling speed of WT or ICAM-2 KO cells compared (Fig. 2D).

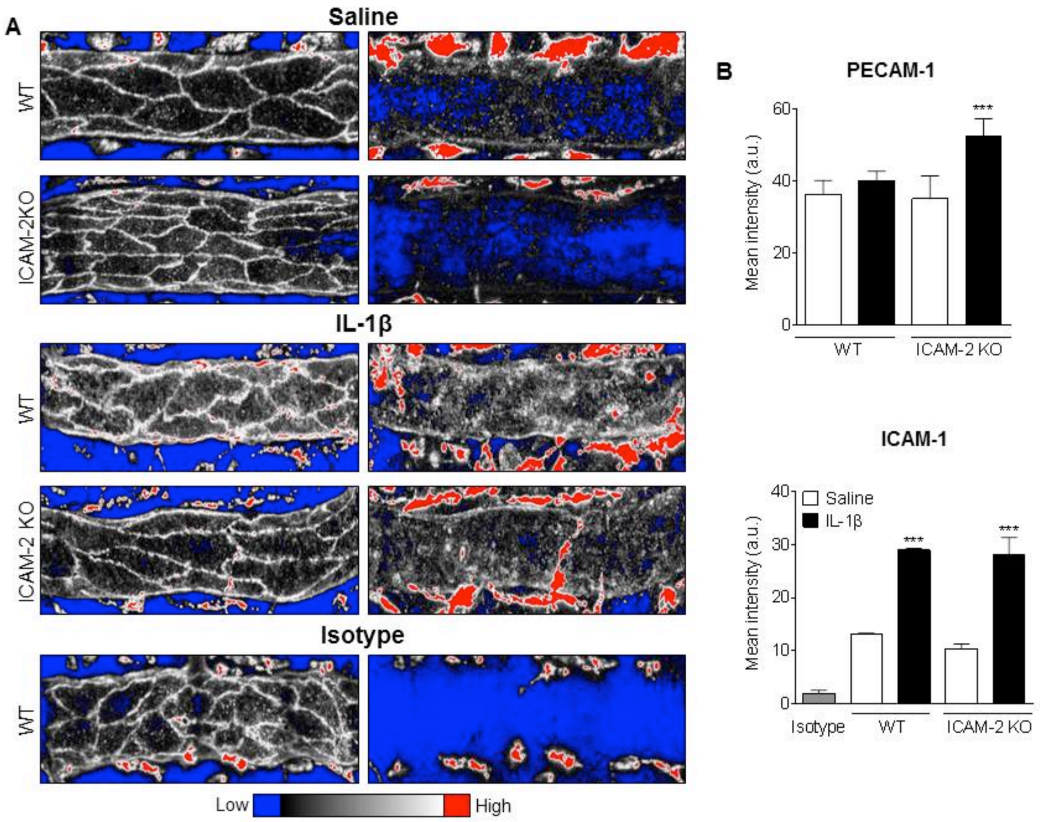

Fig S4. PECAM-1 and ICAM-1 expression in cremasteric post capillary venules. Venular expression of PECAM-1 and ICAM-1 was investigated in WT and ICAM-2 KO mice. Saline or IL-1β-stimulated tissues were analysed by immunofluorescent staining and post-acquisition analysis of total venular expression. (**A**) Representative images of venules from tissues which were immunostained *in vivo* with a (4 µg i.s.) injection of AlexaFluor-647 labelled anti-PECAM-1 mAb and AlexaFluor-555 labelled anti-ICAM-1 mAb (red). The specificity of the ICAM-1 antibody was confirmed by immunolabelling cremaster muscles with isotype control antibody. Scale bar represents 20 µm. The highly stained perivascular structures are cells which non-specifically phagocytose the fluorescently tagged antibodies. (**B**) The total post-capillary venule expression of PECAM-1 and ICAM-1 was quantified in IL-1β (50 ng/mouse) or saline (i.s.) stimulated tissues (4 hours) using Imaris software to build isosurfaces on whole venules based on PECAM-1 expression. N = 3-5 vessels per mice, 3 mice per group, error bars show SEM. Statistically significant (T-test) differences between saline and IL-1β groups are indicated by asterisks, ***P<0.001.

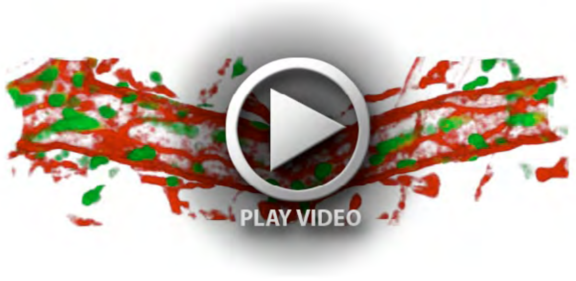

Movie 1. IL-1β stimulated post capillary venule. Shows a cremasteric venule (~40 μm diameter) of a LysM-EGFP$^{+/-}$ mouse, immunostained *in vivo* for EC junctions with AlexaFluor-555-labeled anti-PECAM-1 mAb 390 (4 μg i.s, red) and stimulated with i.s. IL-1β (50 ng i.s.). The movie (captured at ~40X) shows adherent and crawling neutrophils (green) within the vessel lumen at ~2 hrs post injection of the cytokine. Images were captured at one frame per 30 seconds, and the sequence shows a 30 min period.

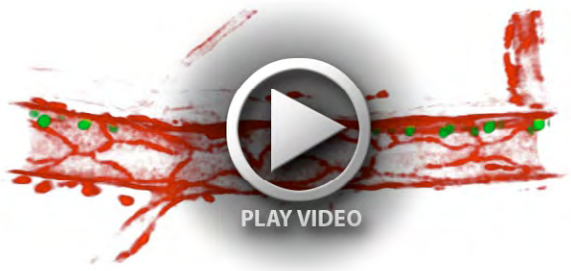

Movie 2. Unstimulated post capillary venule. Shows a cremasteric venule (~40 μm diameter) of a LysM-EGFP$^{+/-}$ mouse immunostained as above, in the absence of any exogenous inflammatory stimulus. The movie (captured at ~40X) shows very few adherent and crawling neutrophils within the vessel lumen, but free flowing neutrophils in the circulation can be seen as a rapidly appearing and disappearing series of spots. Images were captured at one frame per 30 seconds and the sequence shows a 21.5 min period.

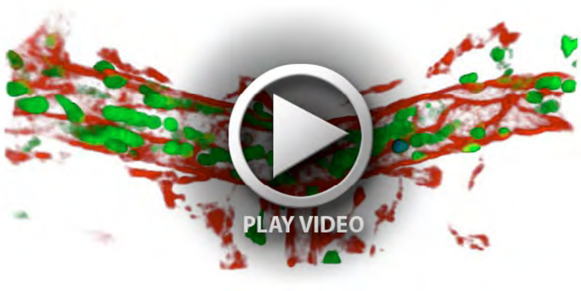

Movie 3. Tracking an individual crawling neutrophil. Shows a cremasteric venule (~30 μm diameter) of a LysM-EGFP$^{+/-}$ mouse immunostained as above, and stimulated with IL-1β (50 ng i.s.). Initially all adherent and crawling neutrophils within the vessel are shown in green, then the green channel is removed leaving just the cell of interest highlighted in blue. The crawling track of this cell is shown as is locomotes across the endothelium, and eventually transmigrates. Images were captured at one frame per 30 seconds and the sequence shows a 26 min period.

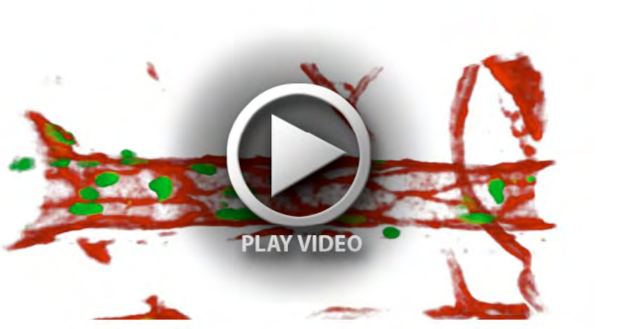

Movie 4. Example of an immobile neutrophil. Shows a cremasteric venule (~35 μm diameter) of a LysM-EGFP$^{+/-}$ mouse immunostained as above, and stimulated with IL-1β (50 ng i.s.). Initially all crawling and adherent neutrophils within the vessel are shown in green, and subsequently the cell of interest is individually highlighted in blue. The cell fails to exhibit motile behaviour for the whole period of observation (32 min), but does show stationary activity in the form of dynamic protrusions, which are quickly retracted.

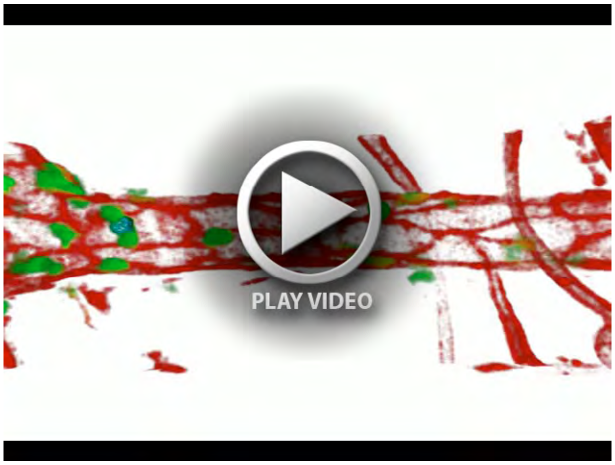

Movie 5. Example of continuous crawling. Shows a cremasteric venule (~35 μm diameter) of a LysM-EGFP$^{+/-}$ mouse immunostained as above, and stimulated with IL-1β (50 ng i.s.). Initially all crawling and adherent neutrophils within the vessel are shown in green, and subsequently the cell of interest alone is highlighted in blue. The cell exhibits continuous crawling behaviour for the entire duration of observation (31 min).

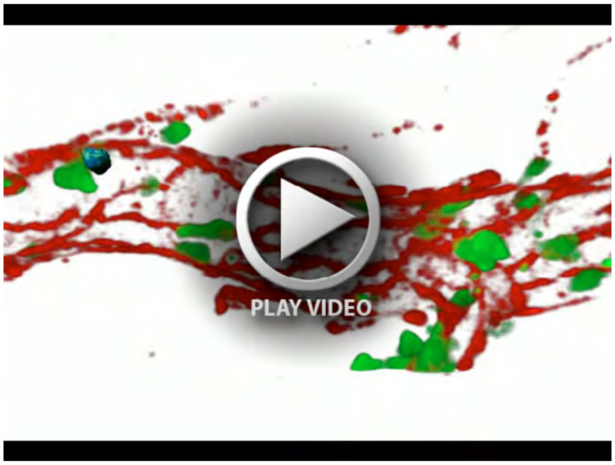

Movie 6. Example of discontinuous crawling. Shows a cremasteric venule (~35 μm diameter) of a LysM-EGFP$^{+/-}$ mouse immunostained as above, and stimulated with IL-1β (50 ng i.s.). Initially all crawling and adherent neutrophils within the vessel are shown in green, and subsequently the cell of interest is individually highlighted in blue. The cell exhibits a period of mobility of ~20 min, then remains immobile and in contact with an EC junction for ~10 min (neutrophil is coloured yellow for this period), before resuming continuous crawling until TEM occurs (38 min in total).

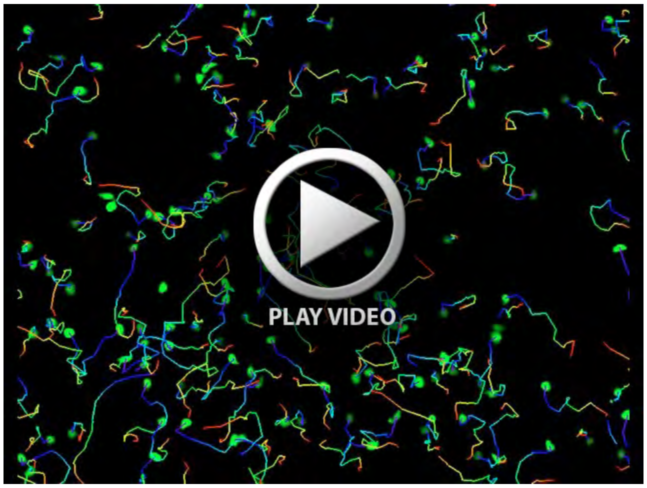

Movie 7. Example of neutrophils crawling on ICAM-2 *in vitro*. Shows purified murine neutrophils, fluorescently labelled with Calcein-AM (green), crawling on ICAM-2 coated glass. Crawling tracks are shown in black. Images were captured at 2 min intervals for 30 min.

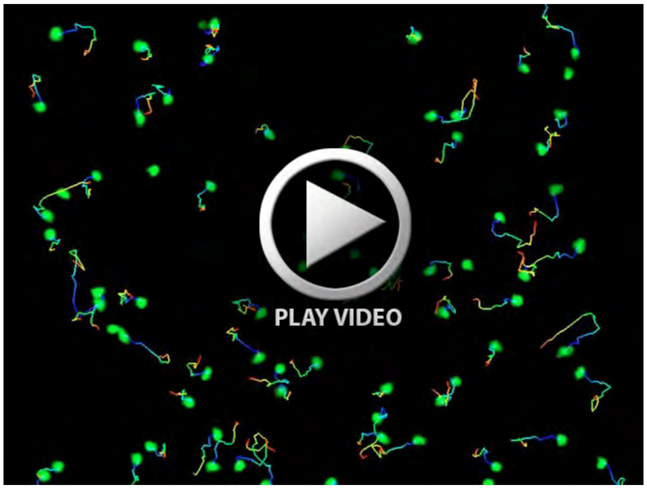

Movie 8. Example of neutrophils crawling on ICAM-2 with anti-MAC-1 treatment *in vitro*. Shows purified murine neutrophils, fluorescently labelled with Calcein-AM (green), crawling on ICAM-2 coated glass, with 10 µg/ml anti-MAC-1 mAb added to the medium. Crawling tracks are shown in black. Images were captured at 2 min intervals for 30 min.

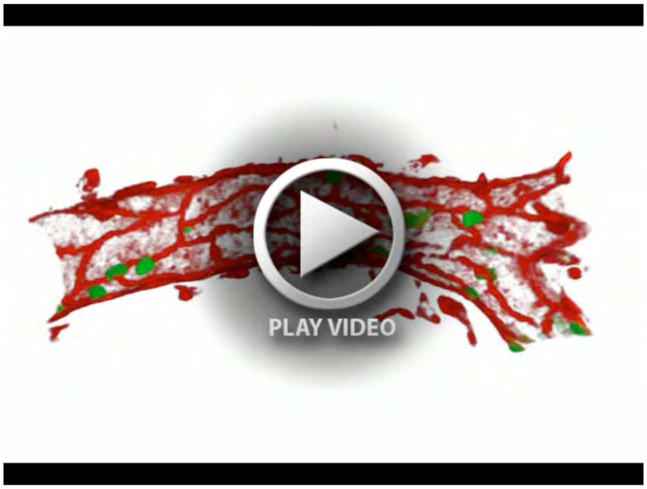

Movie 9. Example of paracellular TEM. The movie shows a cremasteric venule (~50 µm diameter) of a LysM-EGFP$^{+/-}$ mouse immunostained as above, and stimulated with IL-1β (50 ng i.s.). An individual neutrophil can be seen migrating through a paracellular junction between 3 ECs. During the process of transmigration the green channel is switched off in order to illustrate the opening of a paracellular pore.
